# Molecular Characterization of Bovine Leukemia Virus with the Evidence of a New Genotype Circulating in Cattle from Kazakhstan

**DOI:** 10.3390/pathogens11020180

**Published:** 2022-01-28

**Authors:** Akhmetzhan Sultanov, Marzena Rola-Łuszczak, Saltanat Mamanova, Anna Ryło, Zbigniew Osiński, Meruyert A. Saduakassova, Elvira Bashenova, Jacek Kuźmak

**Affiliations:** 1 The Kazakh Scientific-Research Veterinary Institute, Almaty 050016, Kazakhstan; akhmetzhan.sultanov@gmail.com; 2 Department of Biochemistry, National Veterinary Research Institute, 24-100 Puławy, Poland; anna.rylo@piwet.pulawy.pl; 3 Department for Epizootological Monitoring and Risks Assessment of Animal Viral Diseases, The Kazakh Scientific-Research Veterinary Institute, Almaty 050016, Kazakhstan; sal.71@mail.ru (S.M.); mika.kaznivi@gmail.com (M.A.S.); eralievna86@mail.ru (E.B.); 4 Department of Hygiene of Animal Feedingstuffs, National Veterinary Research Institute, 24-100 Puławy, Poland; zbigniew.osinski@piwet.pulawy.pl

**Keywords:** bovine leukemia virus (BLV), BLV proviral load, phylogenetic analysis, genetic variability, Kazakhstan

## Abstract

Bovine leukemia virus (BLV) is a retrovirus that causes enzootic bovine leukosis (EBL) and has worldwide distribution. Infections with BLV have been reported in cattle from Kazakhstan but the virus has not yet been thoroughly characterized. In this study, we detect and estimate the level of BLV proviral DNA by qPCR in DNA samples from 119 cattle naturally infected with BLV, from 18 farms located in four different geographical regions of Kazakhstan. Furthermore, we conducted the phylogenetic and molecular analysis of 41 BLV env-gp51 gene sequences from BLV infected cattle. Phylogenetic analysis showed the affiliation of sequences to two already known genotypes G4 and G7 and also to a new genotype, classified as genotype G12. In addition, a multivariate method was employed for analysis of the association between proviral load and different variables such as the geographical location of the herd, cattle breeds, age of animals, and the presence of particular BLV genotypes. In summary, the results of this study provide the first evidence on molecular characterization of BLV circulating in cattle from Kazakhstan.

## 1. Introduction

Bovine leukemia virus (BLV) is a retrovirus that causes enzootic bovine leukosis (EBL), a neoplastic disease of the lymphatic system in cattle. BLV exhibits a slow, progressive spread within a herd, and it is likely to persist if control measures are not applied. The modes of transmission of the BLV include mainly blood or other body fluids, milk and colostrum feeding by young calves [1,2] and biting flies [3,4]. Most infections are subclinical, but a proportion of cattle (~30%) over 3–5 years old develops persistent lymphocytosis, and a smaller proportion (2–5%) develops lymphosarcomas (malignant tumors) in various internal organs [5].

Infections with BLV are widely distributed worldwide, and many seroepidemiological data proved high prevalence in North and South America, some Asiatic and Middle Eastern countries, and Eastern and Central Europe [6]. The impact of the BLV infection is determined by premature culling of BLV-infected but clinically healthy cattle, production losses due to the reduction in milk production and carcass condemnation at slaughter, and trade restriction of live animals and milk [7,8,9]. In this regard, European countries have implemented eradication programs, based on the detection and sacrifice of BLV-infected animals, according to the OIE rule [10]. However, many other countries where EBL is prevalent do not implement such programs mainly due to high economic costs. Currently, no vaccines or specific treatments are available to control the burden of BLV infection. 

One of the typical characteristics of infection with BLV is the presence of blood cells that carry integrated BLV provirus, referred to as proviral load [11]. Several studies demonstrated that high proviral load may enhance the BLV infection rate [12] and EBL progression [13,14,15]. Recently proviral load was used as a marker to study various factors influencing the course of BLV infection [12,16]. 

Surface glycoprotein gp51, encoded by *env* gene, plays an essential role in BLV infectivity. This protein contains the receptor-binding domain [17], and conformational epitopes, playing a major role in the mounting of neutralizing antibodies [18]. In addition, three neutralization domains, called ND1, ND2, and ND3, were identified to induce BLV-neutralizing antibodies [19]. Gp51 protein contains also the T-cell epitopes (CD4+, CD8+, gp51N5, gp51N11, and gp51N12) involved in cellular immunity to BLV [20]. In this respect BLV *env* gene sequences were the main target for phylogenetic studies, leading to the identification of at least 11 BLV genotypes, distributed worldwide [21,22]. In addition, many studies performed up to date characterized the presence of specific mutations, distributed in the BLV genome, linked to infectivity, replication, and pathogenesis of BLV [21,23,24]. Therefore, there is a strong need to investigate the genetic diversity of BLV, including as many as possible local isolates, from geographically different countries.

The Republic of Kazakhstan is one of the largest Central Asian countries located in the center of Eurasia. It occupies an area of 2,724,900 km^2^. Extensive grazing land and favorable climatic conditions provide a good basis for the development of the livestock sector in which the most important branch is cattle breeding. The character of natural fodder grounds predetermines the development of mostly meat cattle breeding; however, the dairy cattle sector plays a secondary role in ruminant production. In 2021, Kazakhstan owned 9.5 million heads of cattle in total, including 2.5 million dairy cattle, based on the Agency of Statistics of the Republic of Kazakhstan database [25].

In Kazakhstan, the EBL was registered first in early 1966, in two farms in the Karaganda and Almaty regions, among young cattle of brown Latvian and red Lithuanian breeds, imported in 1960 from the Baltic Republics of the Soviet Union [26]. In the years 2015–2019 the serological survey, based on examination of 166,654 cattle by AGID test, showed an average prevalence of 5.7%. The highest prevalence was noted in farms from North Kazakhstan (18.8%), East Kazakhstan (12.1%), West Kazakhstan (14.5%), Kostanay region (14.6%), while regions such as Pavlodar (6.9%) and Zhambyl (4.1%) showed relatively lower prevalence [27]. Molecular analysis of field strains of BLV circulating in cattle from North Kazakhstan was performed using RFLP (restriction fragments length polymorphism) and two subtypes, Belgian and Australian, were identified, with prominent distribution (98%) of Belgian type [28]. 

In the present study, we conducted the phylogenetic and molecular analysis of complete BLV *env*-gp51 gene sequences in DNA extracted from blood samples of cattle from 18 herds, located in different geographical regions of Kazakhstan. In addition, the proviral load was estimated by the use of qPCR and a multivariate statistical method was employed for analysis of the association between proviral load and different variables such as geographical location of the herd, cattle breeds, age of animals and the presence of particular BLV genotypes. Overall, this study provides the first evidence on molecular characterization of BLV circulating in cattle from Kazakhstan.

## 2. Results

### 2.1. Detection of BLV-Infected Cattle by ELISA and qPCR

When all 962 serum samples were tested by AGID and ELISA, 228 (23.72%) were positive by both tests. Serologically positive cattle were found in all 18 farms from six regions and the seroprevalence varied from 0.8% to 84.0%, with the highest rate noted among the farms located in East Kazakhstan (40.0–84.0%) (Table 1). Relatively high seroprevalence was also noted in Kostanay (46.6%), North Kazakhstan (48.8%), and South Kazakhstan regions. However, these data came from single farms only. Moderate seroprevalence (5.0–16.7%) was recorded in farms from the Almaty region, except one farm (ZH) showed 56% seropositive cattle. The lower rate was found in farms from Pavlodar (0.8–3.1%). In order to perform molecular analysis of BLV strains circulating in Kazakhstan, blood samples were collected from seropositive cattle and genomic DNA was extracted from PBLs (peripheral blood leukocytes). We focused on these animals to obtain as many positive results as possible from the amplification of proviral DNA. PBLs fraction was prepared from all 228 seropositive cattle; however, only 186 were subjected to qPCR mainly due to limits in quality and availability of a sufficient amount of DNA. In fact, DNA samples from 119 (63.9%) cattle allocated in 18 farms from all six regions showed the presence of BLV proviral DNA.

### 2.2. Estimation of BLV Proviral Load

Table 1 summarizes the results of proviral load estimation per 1000 cells in animals from all farms. Out of 18 farms analyzed, the highest median value of proviral load (≥100 copies) was noted in seven farms. Two farms, TA and ZH, located in the Almaty region, showed a range between 28.0 and 879.0 copies (median 425.2) and between 1.0 and 481.8 (median 182.7) copies, respectively. Another two farms, KA and KO, from the same region, represented by single animals only, showed the copy numbers 182.9 and 120.7. Two farms, MU (range 1.0–606.0 copies) and KO (range 1.0–542.8 copies), located in North Kazakhstan and Kostanay regions, showed median values of 225.5 and 184.8, respectively. The farm, BO with the range 107.3–487.7 (median 114.3) copy numbers was located in East Kazakhstan. 

Interestingly, only in four farms (KO, MU, BO, and ZH) out of seven the high median values and high ranges of proviral load coincided with relatively high seroprevalence. However, no such relationship was recorded for the four farms in East Kazakhstan, where relatively low copy numbers (median value range 5.2–91.3) corresponded with high seroprevalence, ranging from 40 to 60%. The lowest median value of proviral load (values varied from 1.0 to 40.0 copies), noted in four farms from the Pavlodar region, coincided with the lowest seroprevalence, observed in this study (0.8 to 3.1%).

### 2.3. Relationship between Proviral Load and Variables

**Figure 1:**
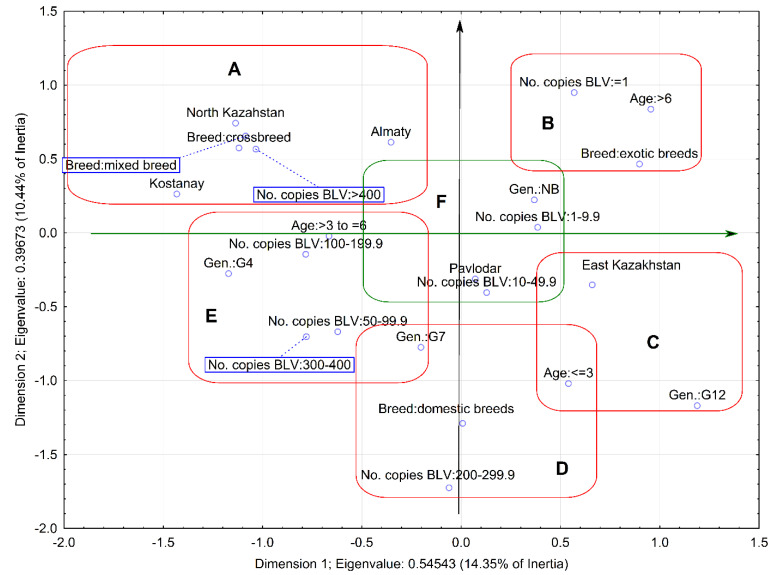
The multivariate analysis (MVA) of factors as breed, age, geographical location of farms, and the genotypes of BLV in correlation to BLV proviral load. Blue points represent each category of analyzed variables. Blue points with similar profile (low value of distances indicating strong association between variables) are marked by the “red oval rectangle” Blue points located in the “green oval rectangle” in the graph’s center showed the points with similar profile but representing eigenvalues indicating lack of any association. “Gen” means genotype while “NB” means samples with known copy number but not subjected for genotyping.

Multivariate analysis was applied on four variables including breed, age, geographical location of farms, and the genotypes of BLV found in the infected cattle with respect to provirus copy number. After plotting all these variables, it was possible to determine discrete differences between proviral load with respect to particular categories of variables within a single graph (Figure 1). Blue points represent each category of analyzed variables. The highest copy number (≥400) created a separated cluster (A), and it was associated with the occurrence of infected cows in two regions—North Kazakhstan and Kostanay—and with infections found in crossbreed (Black and Motley) and mixed breed (Holstein/Black and Motley) cattle (PG = 1.17) (point G). The highest proviral load was also correlated with infection caused by the G4 genotype, although the correlation was not statistically significant (PG = 0.34). Analysis of association between copy numbers varied from 300 to 400, 100 to 199.9, and 50 to 99.9 copies and all variables showed almost identical results and it was therefore treated as homogeneous data in the multivariate analysis. This cluster (E) was characterized by statistically significant association with cattle at an age between 3 and 6 years, an infection caused by the genotype G4 and G7 (PG = 0.83). The occurrence of a copy number between 200 and 300 (cluster D) was significantly correlated with the occurrence of cattle representing ingenious breeds (Alatau, Kazakh white-headed, local without breed), under 3 years of age, and with infections caused by BLV strains belonging to genotype G7 (PG = 1.20). Provirus copy numbers from 1 to 10 and 10 to 50 were associated with animals from regions such as Pavlodar (cluster F). However, multivariate analysis showed no statistically significant association with any of the variables studied (PG = 0.26). If any correlations were observed, they were rather of a random nature. In contrast, proviral load at single copy level was significantly correlated (PG = 1.10) with cattle representing exotic breeds (Simmental and Santa Gertrude), aged over 6 years, as was seen in cluster B. Finally, we identified the last cluster (C) in which a statistically significant correlation (PG = 1.16) was noted between the circulation of genotype G12 and cattle with a different number of copies, at age less than 3 years from East Kazakhstan.

### 2.4. Phylogenetic Analysis Based on ML Method

PCR products of the size of 903 bp were successfully amplified and sequenced from the proviral DNA of 37 BLV isolates. The origin of the samples by region was as follows: Kostanay (8), North Kazakhstan (11), East Kazakhstan (13), and Almaty (5). Unfortunately, amplification of PCR products failed for all six samples coming from the Pavlodar region. In order to keep these samples as representative for this region, nested PCR allowing amplification of 444bp fragments was applied and four samples were successfully amplified. This brings the total number of samples directed to phylogenetic analysis to 41 (Table S1).

**Figure 2:**
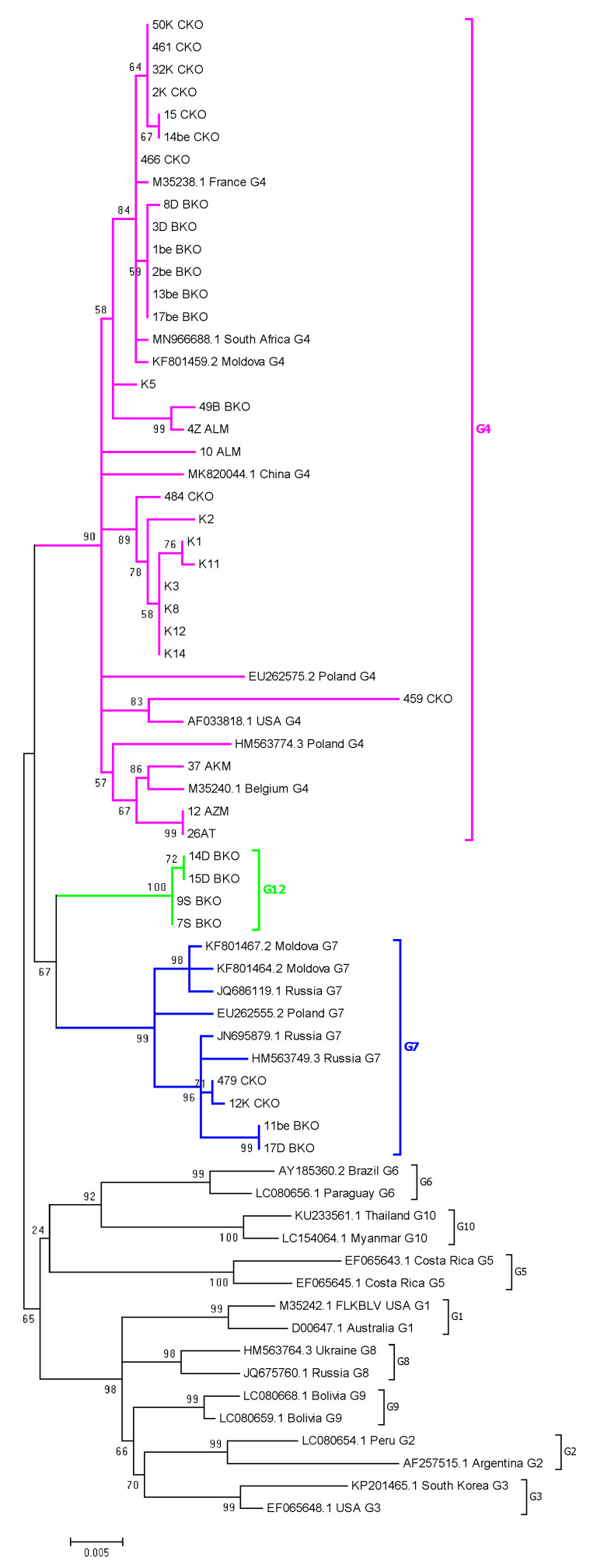
The evolutionary history was inferred by using the maximum likelihood method based on the Kimura 2-parameter model. A discrete gamma distribution was used to model evolutionary rate differences among sites. The tree is drawn to scale, with branch lengths measured in the number of substitutions per site. The analysis involved 67 nucleotide *env* gene sequences 903 bp long. Evolutionary analyses were conducted in MEGA6.

In order to analyze the genetic relationship among BLV isolates from Kazakhstan and those described in the previous studies, a phylogenetic tree based on the ML method was built using 37 BLV sequences, representatives for all regions in Kazakhstan, and 30 other isolates, previously classified within known genotypes G1–G10. Most Kazakhstan isolates (29/37) were clustered to genotype G4, while four belonged to genotype G7, with high bootstrap values of 90% and 99%, respectively (Figure 2). Interestingly, the remaining four isolates, named 7S, 9S, 14D, 15D, from East Kazakhstan, revealed the affiliation to novel G12 genotype, which was supported by 100% bootstrap value (Figure 2). 

Due to the fact that four BLV Kazakhstan isolates coming from the Pavlodar region were available as 400 bp-long sequences only, and because recently identified genotype G11 exclusively represents 423 bp fragment, the ML phylogenetic tree was constructed based on partial *env* gene sequences. This tree included also 42 BLV sequences 400 bp long from different countries. In summary, the phylogenetic data from both trees confirmed the affiliation of 30 Kazakh isolates within genotype G4, seven to genotype G7, and four isolates to a newly identified genotype G12 (Figure S1). 

### 2.5. Assignment of Genotype G12

**Figure 3:**
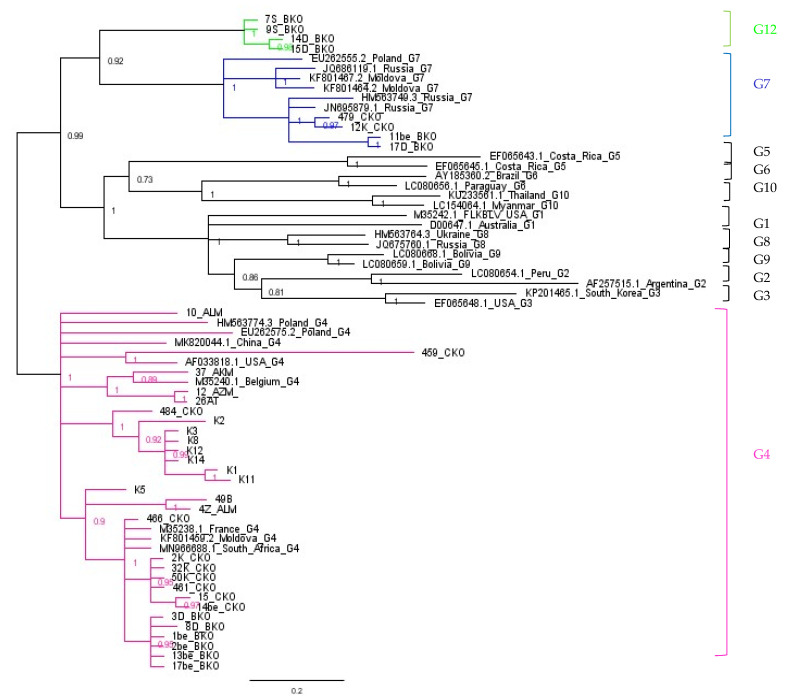
The evolutionary history was inferred by using the Bayesian analysis of the gp51-encoding 903-bp fragment of the *env* gene nucleotide sequences. Numbers at nodes indicate posterior probabilities of sampling the node among 11,000 trees. Genotypes and subtypes as well as a new genotype found in this study are indicated at the right by vertical lines.

To confirm the data from ML analysis showing the affiliation of four sequences to the new genotype G12, Bayesian interference (BI) was performed using MrBayes with the GTR substitution model and 37 BLV sequences already classified by ML tree (Figure 3). The ML and BI trees showed congruent results. The MrBayes tree topology indicated that four of Kazakhstan BLV strains cluster into a unique clade with a high posterior probability of value 1.00, which confirmed the presence of a new genotype G12 (Figure 3). In addition to confirming whether these four isolates belonged to the new G12 genotype, the mean genetic distances within and between BLV genotypes G1–G10 and G12 were calculated (Table 2), using data available from GenBank. Sequences were downloaded from NCBI during July 2021. In order to identify BLV sequences the following search terms were used: ((“Bovine leukemia virus”[Organism] OR Bovine Leukemia Virus[All Fields]) AND env[All Fields] AND (“Bovine leukemia virus”[Organism] OR BLV[All Fields])) AND (“903”[SLEN]: “9000”[SLEN]). Sequences representative for genomic DNA from *Bos taurus* were exclusively included in this analysis. Then, the sequences were manually inspected, aligned, and cropped to 903 bp using BioEdit software [29]. Sequences that have been assigned to the particular genotype at least one time were used. This resulted in 254 original sequences ( including genotype G12) listed in Table S2. As was shown in Table 2 the mean distances between genotype G12 and other genotypes varied from 2.5% to 4.0%, and these values were higher than the lowest value of 1.9% quoted between G8 and G9. Furthermore, the mean genetic distances between genotype G12 and other genotypes were higher than the mean intragenotype distances, within each of the analyzed genotypes.

### 2.6. Comparison of Nucleotide Sequences Belonging to Genotype G4, G7 and G12 

**Figure 4:**
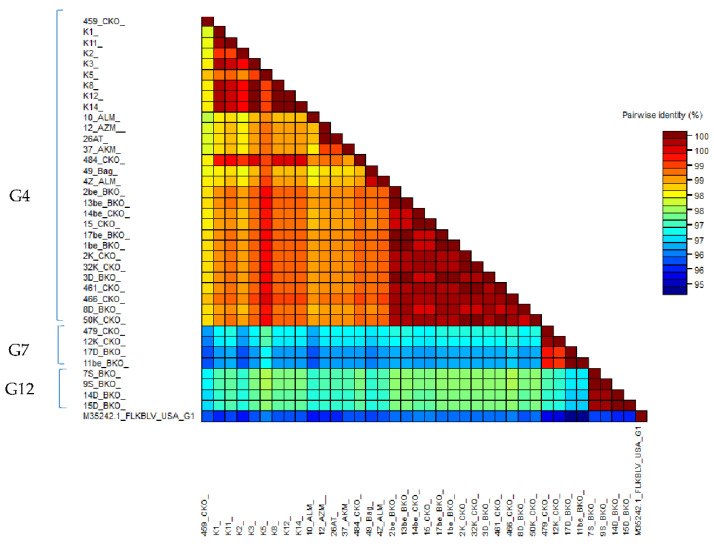
SDT color-coded matrix of pairwise identity scores generated by the alignment of a G4, G7 and G12 903 bp long BLV *env* gene set of nucleotide sequences for 37 Kazakh BLV isolates and FLK-BLV strain.

Pairwise comparison of 37 of 903 bp-long sequences and 41 of 400 bp-long sequences obtained from Kazakhstan and the FLK-BLV sequence, representative for genotype G1, was carried out. The identity scores were represented as color-coded blocks using SDT v.1.2 software [30] (Figure 4) and numerical values are shown in excel files (Table S3). Generally, the sequence identity among 903 bp-long sequences varied between 96.3–100%. Pairwise identity of sequences representing genotypes G4, G7, and G12 ranged from 98.2 to 100%, 99.2 to 100%, and from 99.9 to 100%, respectively. When pairwise comparison was extended over the four sequences, 400 bp long, generally the sequence identity was similar to those noted for longer fragments and varied between 96.8 and 100%. In particular, the identity varied between 98.3–100% for genotype G4, 99.3–100% for G7, and 99.8–100% for new genotype G12 (Figure S2; Table S3).

### 2.7. Amino Acid Sequence Analysis

**Figure 5:**
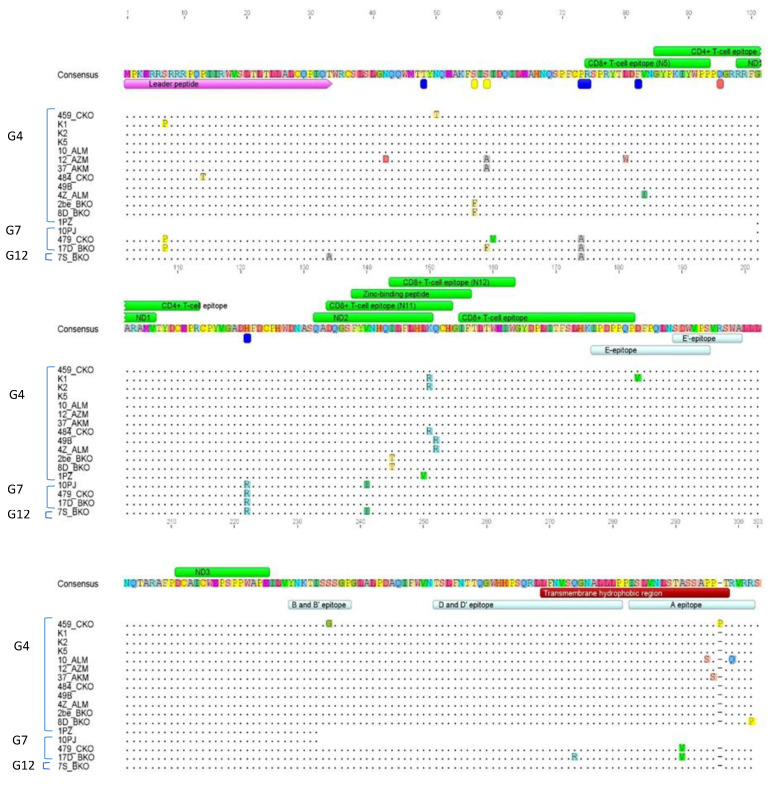
Alignment of the deduced amino acid sequences of whole gp51 protein of representative seventeen Kazakh BLV strains. Differences from the consensus sequence are indicated as is the distribution of corresponding antigenic determinants. Horizontal bars indicate the leader peptide, CD8+ and CD4+ T cell epitopes, zinc-binding peptide and the antigenic determinants epitopes E, E’, B and B’, D and D’, A (linear), F, G, H (conformational, red, blue and yellow bars, respectively), ND1, 2, 3—neutralization domain, and the TMHR transmembrane hydrophobic region.

Figure 5 shows the alignment of 17 deduced amino acids sequences which were distinct among 41, originally used for the analysis. A total number of 27 aa substitutions were found in these sequences. Three substitutions (S7P, P13T, and T33A) were localized at signal peptide, three (S56F and S58A or S58F) were found in epitope H and two (P73A and H121R) in epitope G. Two substitutions ( L80W, V83I) were localized at CD8+ T-cell epitope (N5), next four (V140I, I144T, L149V, and K150R was seen in the region spanning neutralizing domain 2 (ND2), CD8+ T-cell epitope (N11 and N12) and Zinc-binding peptide and one more Q151R were situated just behind ND2 domain. The remaining eight substitutions were localized in linear epitopes, namely D183V in epitope E-E’, S235G in B-B’ epitope, Q274R in DD’ epitope, and finally, substitutions A291V, A295S, P296S, R298Q, and R301P were found in epitope A. Three substitutions N42D, N50T, and I59V were found outside the specific domains of the gp51 protein. Among 27 aa substitutions, four (P13T, N42D, N50T, R298Q) were found to be unique for Kazakhstan aa sequences. The remaining 23 substitutions were earlier identified among BLV isolates worldwide. Interestingly, nucleotide sequence analysis of sample 459_CKO showed an insertion of three nucleotides (CTC) located at position 881 in the transmembrane hydrophobic region/A epitope. Despite this fact, this insertion did not cause the frameshift mutation but resulted only in the insertion of an additional amino acid (proline) at position 297 (Figure 5).

Since genotype G12 was described as a new BLV genotype, we focused on analysis of aa changes specific for this genotype. When comparing G12 amino acids sequences to 250 aa sequences, representative for known G1–G10 genotypes (Table S4) we found that substitution of threonine by alanine at position 33 (T33A) was specific for genotype the G12. This comparison showed also that substitution alanine by valine at position 291 (A291V) was differentiating genotype G12 from G7. Such changes at aa sequences make it likely that the genotype G12 is present as a distinct genotype. This conclusion was confirmed by phylogenetic analysis based on aa sequences (Figure S3).

## 3. Discussion

The present study investigated the molecular nature of BLV infection in cattle from Kazakhstan. We focused on the following two aspects of such analysis: (I) the detection of BLV proviral DNA and its association load with various factors under field conditions, (II) phylogenetic and molecular analysis of field isolates of BLV. The effort was firstly directed on the identification of cows infected with BLV as determined by ELISA and qPCR. Serum samples collected from cattle from 18 farms located in different geographical areas showed the presence of seropositive animals in all of them, suggesting that BLV infection might be quite common in Kazakhstan. The data showed also different seroreactivity with the highest values noted in East Kazakhstan (40.0–84.0%), North Kazakhstan (48.8%), and Kostanay (46.6%) regions, moderate in the Almaty region (2.5–16.7%) and remarkably low (0.8–3.1%) in the Pavlodar region. Differences in the seroreactivity to BLV are likely to occur among different regions of the same country and they are influenced by a number of factors, as was recently noted in Vietnam [31], Myanmar [12] and Egypt [16]. The results of this study concurred with previous seroepidemiological studies in Kazakhstan [27]. showing a similar distribution of BLV seroreactivity between particular regions. BLV infection was also found in the neighboring countries and includes 28.5-36.1% in Russia [32], 3.9% in Mongolia [33] and 49.1% in China [34].

Next, the proviral load was estimated in DNA samples from 119 cattle, and this is the first study elucidating the presence of proviral DNA in cattle naturally infected with BLV in Kazakhstan. Several studies documented that estimation of copy number of BLV proviral DNA integrated with genomic DNA in the host cells has a prognostic value for the development of EBL [13,35]. It was also shown that BLV proviral load in the infected animals reflex a potential of virus transmission since the removal of cattle with high proviral load was successful in reducing BLV prevalence and incidence [36]. In contrary, cattle with low proviral load appear to be less important in the transmission of BLV infections [37,38]. It is worth mentioning that the proviral load in an individual cattle is not stable and fluctuates over the course of BLV infection [39]. 

In the present study, we noted a prominent variation in proviral copy numbers between animals in particular farms as well as between animals from different farms. BLV positive cattle from seven farms in three regions (Almaty, Kostanay, and North Kazakhstan) exhibited the highest median value of proviral load, while the cows from farms located in Pavlodar and East Kazakhstan showed lower proviral concentration. Furthermore, this study showed that the median value of proviral load did not fully correlate with the BLV seropositivity, since only in four farms did the high proviral load coincide with relatively high seroprevalence. A direct association between proviral load and the seroprevalence could be expected because a high proviral load determines a high level of infection and a greater transmission probability [40]. However, such argumentation cannot be used to explain the results recorded in four farms from East Kazakhstan, where low copy numbers corresponded to relatively high seroprevalence, varying from 40 to 60%. Therefore, this could indicate that the variation of the proviral load in BLV-infected cows from Kazakhstan could be due to different factors. 

Using multivariate analysis, we assessed the association between proviral load and variables such as geographical location of farms, breed, age, and BLV genotypes. This analysis clearly showed that the high proviral load in cattle, especially those noted in North Kazakhstan and Kostanay, was associated with infections found in crossbreed (Black and Motley) and mixed breed (Holstein/Black and Motley) cattle. The maintenance of these breeds in North Kazakhstan and the Kostanay regions is quite frequent and offers many positive aspects linked to their high productivity and the possibility to withstand quite harsh and unfavorable climatic and weather conditions. Since Holstein cattle are known to be highly susceptible to BLV [1,41] we consider that housing of mixed breeds could be the factor promoting the transmission of BLV. High proviral load was also found in cattle representing ingenious breeds such as Alatau and Kazakh white-headed, suggesting their high susceptibility to BLV. However, such results contradict previous observations from Myanmar and Vietnam documenting lower susceptibility of the native breed to BLV infection [12,31]. Multivariate analysis showed that low proviral load noted in cattle from Pavlodar and East Kazakhstan was significantly associated with cattle representing exotic breeds such as Simmental and Santa Gertrude. Collectively, our observation indicates that variations in proviral load may be explained by the association between breed and genomic factors such as polymorphism of BoLA-DRB3 gene [35,42,43]. 

BLV induces persistent infections in cattle, and it is expected that the older animals have been infected for a long period. Therefore, a tendency toward higher prevalence was noted in older animals [44,45,46]. We should also expect the progressive accumulation of proviral copies in persistently infected animals. However, in this study, we were unable to demonstrate an association between proviral load and age, since the cattle with high proviral load were under 6 years old, while the cattle with single-copy levels were aged over 6 years. Similarly, age was recognized as a factor influencing only limited association of proviral load [14]. 

The phylogenetic analysis based on complete and partial env-gp51 gene sequences, and two different tree-building methods identified tree BLV genotypes (G4, G7, and G12) circulating in cattle from Kazakhstan. Genotype G4 was the most prevalent genotype and was detected in 9 farms out of 13 subject to phylogenetic study, while genotype G7 was found in 6 farms. Newly detected genotype G12 was identified in two farms from East Kazakhstan. Interestingly, the presence of genotypes G4/G7 in the same herd was found in two farms from North and East Kazakhstan, and the presence of all three genotypes G4/G7/G12 were registered in one farm from East Kazakhstan. The data from the multivariate analysis showed that no correlation was noted between proviral load and infection caused by G4. However, a statistically significant association was also noted between genotype G7 and proviral concentration at the level of 200–300 copies. The presence of genotype G12 in two farms was linked to different concentrations of the provirus. These results can suggest that there is no clear indication that specific genotypes of BLV are associated with provirus load. 

For molecular epidemiology of BLV infection in Kazakhstan, it is interesting to determine the origin of three different BLV genotypes identified in this study. During the last two decades, there have been significant changes in dairy cattle breeding in Kazakhstan, due to the rapidly growing demand for milk and dairy products and also due to the development of dairy industries. To meet these needs, since the beginning of the 2000s, Kazakhstan has been importing large numbers of cattle of exotic breeds such as Holstein-Frisian, Hereford, Simmental from such countries as the Russian Federation, Germany, Ukraine, Canada, and USA [47]. The Simmental breed is distributed rather unevenly but the large livestock of this breed is located in the eastern part of the Republic of Kazakhstan [48]. Black and Motley breed was imported to Kazakhstan from the Russian Federation many decades ago for the development of local dairy initiatives and herds with this breed are located mainly in the north part of the country. The presence of the genotypes G4 and G7 was reported for some European, North and South American countries, and China [21,49,50]. Both genotypes were also identified in the infected cattle from Western Siberia and Mongolia [33,49]. Since the virus’ introduction to the herd through the import of infected animals is considered as the main route of BLV transmission [1,51,52] it is clear that the affiliation of BLV strains from Kazakhstan within G4 and G7 genotypes resulted in extensive cattle trading in the past. Indeed, it took place between the former republics of the Soviet Union and countries belonging to the Council for Mutual Economic Assistance (CMEA), in the second half of the 20th century. Likewise, the import of live animals and frozen bull semen took place from North America where infection with BLV are known to be present [7] and where the presence of genotype G4 was confirmed [53]. Although genotype G4 and G7 were also found in some South American countries, there is no official information on live cattle trade between them and Kazakhstan. Surprisingly, none of the strains from Kazakhstan clustered in genotypes G1, G6, G10, and G11, the genotypes that were recently identified in neighboring countries Mongolia and China and that was thought to be specific for this geographical area [22,33,54]. Genotype G11 was found in Heilongjiang province and genotypes G6 and G10 in yaks from Qinghai-Tibet Plateau and both places are located very far from Kazakhstan. Genotype G1 was found in cattle from Mongolia, but there was no cattle trade to Kazakhstan from this country. 

We also reported the specific topology of the phylogenetic tree characterized by the presence of several subgroups, within genotypes G4, G7, and G12, which was supported by significant branch support. These subgroups included the strains grouped according to the similar place of origin. We noted the same features of genotypes G4 and G7 found in our previous study on BLV isolates from Eastern Europe and Siberia [49] and Moldova [55]. Thus, we could conclude that region-tailored subgrouping of BLV strains occurs as a result of the dissemination of a fraction of diverse virus populations which subsequently, over time, become homogenous. This remains consistent with the concept of the importance of geographically distinct BLV isolates in the global diversification of BLV [56].

The most exciting finding in this study was the identification of a new genotype G12. This finding was supported not only by ML and Bayesian analysis but also by intra- and inter-genotype genetic distance analysis. Up to now, eleven genotypes (G1–G11) were identified [22] and the appearance of a new genotype should be expected as a result of the continuous evolution and global diversity of BLV. Sequences belonging to G12 come from two farms, SR and DO, located in East Kazakhstan, quite far from each other, being without any epidemiological links. G12 genotype was the unique one infecting cattle in farm SR, while infection with G12 in farm DO were accompanied by infections with G4 and G7 genotypes. The presence of more than one genotype of BLV in one farm or certain regions have been reported previously [55,57]. We suppose that the BLV infected cattle were introduced into these farms in the past and subsequently the infection was established, since any eradication program was not implemented. In fact, both farms showed a high seroprevalence of BLV, close to 60%. Our hypothesis is that the viruses belonging to genotype G12, circulating at least in farm DO, would emerge from genotype G7. In our study, genotype G12 comprised sequences clearly distinguished on the phylogenetic trees and supported by significant branch support (posterior probabilities from 0.98 to 1.00 and bootstrap values from 72% to 100%). Since isolates belonging to G12 genotype shared a common node with genotype G7, we suggest that genotype G7, but not G4, would be their common ancestor. This was confirmed by low value of evolutionary divergence (2.7%) when both clusters of G7 and G12 sequences were compared to each other. 

In total, 27 amino acid substitutions were found in this study in epitopes or functional domains of gp51 such as epitopes G and H, CD8+ T-cell epitope, neutralizing domain 2 (ND2), CD8+ T-cell epitope (N11 and N12), and Zinc-binding peptide, not at a random location. Interestingly, 86% of all amino acid substitutions reported here have been described previously [12,21,58,59]. Their identification in the analyzed *env*-gp51sequences indicates that the sequences of the genotype G7 were more conserved than those of the genotype G4. Four substitutions (P13T, N42D, N50T, R298Q) were found to be unique for Kazakhstan aa sequences. What should be highlighted is that substitution T33A was typical of the newly identified G12 genotype.

In conclusion, this study showed for the first time, phylogenetic analysis of the BLV *env* gene sequences in cattle from Kazakhstan, with their subsequent molecular analysis. Thus, these investigations complement the major gap of BLV research, which is the limited number of available viral sequences, representing geographically diverse local strains. Furthermore, this study provided topical data on the epidemiology of BLV infection in Kazakhstan, including risk factor analysis, which will help develop and implement effective plans of BLV control in Kazakhstan.

## 4. Materials and Methods

### 4.1. Sample Collection and DNA Extraction

**Figure 6:**
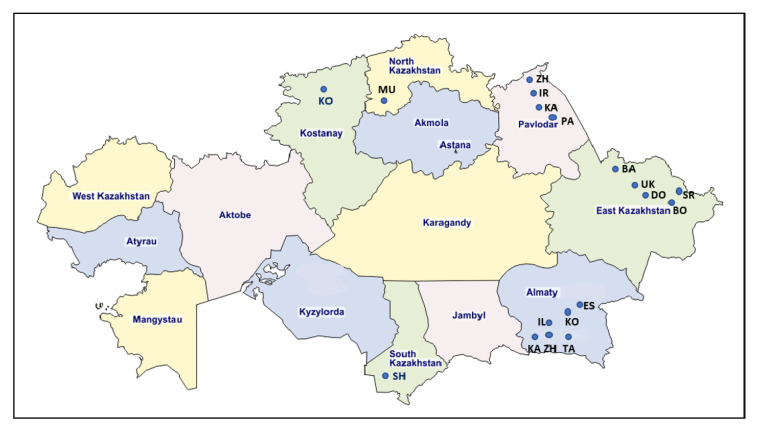
Map of Kazakhstan showing the location of regions and farms, from which samples were obtained for testing. Adapted map of Kazakhstan originally comes from https://d-maps.com (accessed on line 13 January 2022).

Blood samples were collected from 962 cows randomly selected from 18 industrial dairy farms, located in the following 6 regions of Kazakhstan: Kostanay, North Kazakhstan, Pavlodar, East Kazakhstan, Almaty, and South Kazakhstan (Turkistan) (Table 1 and Figure 6). 

These farms were randomly selected taking into consideration the regions with prominent production of dairy cattle in the country and the presence of seropositive positive animals, based on a previous survey. All sampled animals belonged to different breeds, such as exotic breeds (Simmental and Santa Gertrude), crossbreeds (Black and Motley), mixed breeds (Holstein/Black and Motley), and ingenious breeds (Alatau, Kazakh white-headed, local without breed), at ages varying from 1.5 to 14 years. Serological testing was performed using AGID test (BIOK, Kursk Biofactory, Russia) and ELISA (IDEXX Leukosis Serum X2 Ab Test, IDEXX, Liebefeld-Bern, Switzerland). Peripheral blood leukocytes (PBLs) were isolated from blood samples by centrifugation at 1500 g for 25 min and erythrocytes were hemolyzed by osmotic shock with H_2_O and 4.5% NaCl. After two washes in PBS, the supernatant was discarded, and the cells were kept as dry pellets. This part of the work was carried out in the Laboratory of Virology of the Kazakh Research Veterinary Institute (KazSRVI LLP, Almaty, Kazakhstan). Next, the PBLs pellets were sent to the National Veterinary Research Institute in Pulawy, Poland for DNA extraction and further molecular analyses. The genomic DNA was extracted using NucleoSpin Blood Kit (Macherey Nagel GmbH & Co KG, Dueren, Germany), according to the manufacturer’s recommendation. The quality and quantity of DNA was evaluated in a Nanophotometer (Implen GmbH, Munich, Germany).

### 4.2. Proviral Load Quantification

The BLV qPCR was performed as was previously published [60]. The reaction mixture included 12.5 µL of 2× QuantiTect Multiplex PCR NOROX Master Mix (Qiagen, Hilden, Germany), 0.4 µM of each of the primers, 0.2 µM of BLV probe and 500 ng of genomic DNA, in a total volume of 25 µL. The amplification was performed in the Rotor-Gene Q System (Qiagen, Hilden, Germany) using an initial denaturation step and polymerase activation at 95 °C for 15 min, followed by 50 cycles of 94 °C for 60 seconds and 60 °C for 60 s. Ten-fold dilutions of pBLV1 plasmid containing 120 bp fragment of pol gene were made from 1 × 10^0^ to 1 × 10^5^ copies per reaction and used to construct standard curve and estimate the BLV copy numbers. To measure provirus copy number per 1000 cells bovine histone H3 family 3A (H3F3A) gene was amplified by qPCR and ten-fold dilutions of the pDNA from 10^2^ to 10^6^ copies per μL were used to construct a standard curve [61]. The qPCR was accomplished in a 25 μL final volume containing a mixture of 12.5 μL 2× QuantiTect Multiplex PCR NoROX master mix (Qiagen, Hilden, Germany), 0.4 μM of each primer (Genomed, Warsaw, Poland), 0.2 μM probe, and 500 ng genomic DNA in the Rotor-Gene Q cycler (Qiagen, Hilden, Germany). The number of BLV provirus copies per 1000 cells was calculated as follows: (copy number of BLV pol)/(copy number of H3F3A/2) × 1000.

### 4.3. PCR Amplification of 993 bp and 444 bp Fragments of Env Gene

A semi-nested PCR was employed to amplify the 993 bp fragment of env gene. The reaction was performed using high-fidelity PrimeSTAR Max DNA Polymerase (Takara Bio, Kyoto, Japan) and forward primer AP_4762 (5′-GCTCTCCTGGCTACTGACC-3′) [55] and reverse ones ZM2 (5′-TCTGATGGCTAAGGGCAGACACGGC-3′) and ZM5 (5′-GCTAG GCCTAAGGTC AGG GCCGC-3′) for nested PCR, respectively [62]. The reaction mixture contained 200 ng of DNA, 25 μL of PrimeSTAR Max DNA premix, 1 μL of each primer (2.5 μM) and 2.5 units of high-fidelity PrimeSTAR Max DNA Polymerase). For nested PCR 5 μL of initial PCR mixture was used. For both PCRs the same thermal conditions were used, as follows: 10 s at 98 °C followed by 30 cycles of 10 s at 98 °C, 5 s at 55 °C, and 10s at 72 °C. The primers for nested PCR amplification were described previously by Fechner et al. (1997) and their sequences were as follows: env 5032 (5′-TCTGTGCCAAGTCTCCCAGATA-3′); env 5608 (5′-AACAACAACCTCTGGGAAGGGT-3′) and env 5099 (5′-CCCACAAGGGCGGCGCCGGTTT-3′), env 5521 (5′-GCGAGGCCGGGTCCA GAGCTGG-3′). Amplification was performed with 500 ng of genomic DNA using Thermal Cycler (Biometra) with the following cycling conditions: 2 min at 94 °C, 30 s at 95 °C, 30 s at 62 °C (external primers), or 30 s at 70 °C (internal primers), 1 min at 72 °C; after the last (40th) cycle, the samples were incubated at 72 °C for 4 min. Each 50 mL reaction contained 5 mL 10× buffer, 1 mL of 10 mM dNTPs, 1.5 mL of 10 mM of each primer and 2.5 units of DreamTaq DNA Polymerase (Thermo Scientific, Vilnius Lithuania). Nested and semi-nested PCR products were separated and analyzed by electrophoresis on 1.5% agarose gel containing SimplySafe (EURX, Gdańsk, Poland) in 1× TAE buffer.

### 4.4. DNA Sequencing and Sequence Analysis

PCR products were purified and sequenced in both directions by the Genomed SA Company (Warsaw, Poland), using a 3730xlDNAAnalyzer (Applied Biosystems, Foster City, CA, USA) and a BigDye Terminator v3.1 Cycle Sequencing Kit (Life Technologies, Carlsbad, CA, USA ). The sequence data were edited and aligned using the Geneious Alignment module within Geneious Pro 5.3 Software (Biomatters Ltd., Auckland, New Zealand) [63]. The resultant sequences representing 400 bp and 903 bp fragments after subtracting the length of the primers and additional nucleotides for 903 bp fragments to obtain sequence coding signal peptide and gp51 protein, were then submitted to the GenBank database and assigned accession numbers, as documented in Table S1. In addition, sequences representative for known BLV genotypes G1–G11 were also included in this analysis (Table S1). For robust and accurate phylogenetic analysis of the env sequences, phylogenetic trees were constructed using two different algorithms. Phylogenetic analysis was conducted using MEGA 6 software [64]. The Kimura-2 parameter model with gamma distribution (K2+G) was chosen as the model with the best fit for accurate phylogenetic analysis of 903 bp and Kimura-2 parameter model of 400 bp sequences, using the “find best DNA/Protein models” tool of MEGA 6 software. The reliability of the phylogenetic relationships was evaluated by nonparametric bootstrap analysis with 1000 replicates.

Next, to confirm the data obtained by ML analysis, BI was performed using MrBayes with the GTR substitution model within Geneious Pro 5.3 Software (Biomatters Ltd, Auckland, New Zealand) [63]. Estimates of evolutionary divergence over sequence pairs between genotypes and within genotypes were calculated using the MEGA 6 software application [64] according to the p-distance substitution model. Phylogenetic tree of gp51 amino acids sequences was inferred by using the Maximum Likelihood method based on the JTT matrix-based model (MEGA6).

A pairwise identity matrix of sequences belonging to Genotype G1 (903 bp and 400 bp-long sequences) were inferred using Sequence Demarcation Tool Version 1.2 (SDTv1.2) software (Computational Biology Group, Cape Town, South Africa) [30]. Deduction of amino acid sequences through the translation of nucleotide to amino acid sequences was performed using Geneious Pro 5.3 Software (Biomatters Ltd., Auckland, New Zealand) [63].

### 4.5. Multivariate Analysis

The multivariate (MVA) statistical analysis [65] was used for the identification of any association between BLV provirus copy number and different categories of variables. The analysis was undertaken using individual data from all 119 cattle that were explored for variables such as breed, age, and geographical location of farms, while the data collected from 37 cattle were used for genotype analysis. Provirus copy number was scored at the following ranges: single copy, 1–9.9, 10.0–49.9, 50.0–99.9, 100.0–199.9, 200.0–299.9, 300.0–399.9, 400, and more. MVA data were analyzed using Statistica software, version 10.0 (StatSoft Inc, Tulsa, USA) and *p* ≤ 0.05 was considered as statistically significant. In the Statistica software, computed tables included columns with copy number and variables such as breed, age, and geographical location of farms as well as particular genotypes, while the rows included the data (copy number, breed, age, genotype) that corresponded to the particular animal. For each dimension and row or column point, the software computed the statistical parameters of MVA such as inertia, quality, and eigenvalues [66,67]. Based on this analysis, the above mentioned coordinates, were compiled in a two-dimensional graph. The distances between any row points or column points give a measure of their similarity or dissimilarity. Points grouped around their respective coordinates formed a given cluster, which was graphically marked on the graph. Statistically significant relation between copy numbers and particular variables was calculated and expressed as PG (point G) values. PG values close to zero indicated the lack of statistically significant relations, while values higher than 0.5 showed the presence of a statistically significant relation.

## Figures and Tables

**Table 1:** BLV detection results examined using qPCR and PCRs targeting *env*-gp51 region followed by sequencing.

Region	Farm ID	Breed	Age (y.m)	Seropositive No./ Tested No. (Positive %)	Positive No./Tested No. by qPCR	Number of Provirus Copies Per 1000 Cells (Range, Median Value )	Number of Sequences 903 bp	Number of Sequences 444 bp	Genotypes
Kostanay	KO	Black and motley	3.6–5.0	14/30 (46.6)	14/14	1.0–542.8 184.8	8	0	G4 (8)
North Kazakhstan	MU	Black and motley	5.0–11.0	43/88 (48.8)	17/17	1.0–606.0 225.5	11	0	G4 (9) G7 (2)
Pavlodar	IR	Simmental	4.0–5.0	2/64 (3.1)	2/2	1.0–2.3 1.0	0	1	G7 (1)
	ZH	Kazakh—white-headed	4.0	1/125 (0.8)	1/1	1.2	0	1	G4 (1)
	PA	Simmental	4.0–5.0	2/120 (1.7)	2/2	1.0–45.1 22.8	0	1	G7 (1)
	KA	Kazakh—white-headed	5.0	1/65 (1.5)	1/1	40.0	0	1	G7 (1)
East Kazakhstan	BA	Simmental	1.6	20/50 (40.0)	12/16	1.0–66.2 17.6	1	0	G4 (1)
	UK	Simmental	5.0–10.5	27/50 (54.0)	16/20	1.0–348.1 87.3	0	0	0
	SR	Simmental	6.0–14.1	19/30 (63.3)	12/19	1.0–18.4 5.2	2	0	G12 (2)
	DO	Alatau/Local, without breed	2.0–3.9	15/25 (60.0)	13/14	11.5–367.7 91.3	5	0	G4 (2) G7 (1) G12 (2)
	BO	Local, without breed	2.1–4.9	21/25 (84.0)	14/19	107.3–484.7 114.3	5	0	G4 (4) G7 (1)
Almaty	ES	Local, without breed	6.0–7.4	2/40 (5.0)	0/2	0	0	0	0
	ZH	Santa Gertrude	4.0–6.0	28/50 (56.0)	10/23	1.0–481.9 182.7	3	0	G4 (3)
	TA	Holstein/ Black and motley	4.0	5/30 (16.7)	2/3	28.0–879.0 425.2	1	0	G4 (1)
	KA	Local, without breed	6.0	1/40 (2.5)	1/1	182.9	0	0	0
	KO	Local, without breed	5.0	4/40 (10.0)	1/2	120.7	1	0	G4 (1)
	IL	Black and motley	5.0	3/20 (15.0)	1/3	1.0	0	0	0
South Kazakhstan	SH	Local, without breed		27/40 (67.5)	0/27	0	0	0	0
Total	18			228/962 (23.7)	119/186 (63.9)		37	4	G4 (30) G7 (7) G12 (4)

**Table 2:** Estimates of evolutionary divergence over sequence pairs between genotypes and within genotypes. The number of base differences per site from averaging over all sequence pairs between groups are shown with standard errors. The lower matrix shows intergenotype nucleotide evolutionary divergences and diagonal columns show intragenotype nucleotide evolutionary divergences. The analysis involved 254 *env* gene nucleotide sequences. Codon positions included were 1st + 2nd + 3rd + Noncoding. All ambiguous positions were removed for each sequence pair. There were a total of 903 positions in the final dataset. Evolutionary analyses were conducted in MEGA6.

	G1	G2	G3	G4	G5	G6	G7	G8	G9	G10	G12
**G1**	0.008 (±0.001)										
**G2**	0.030 ± 0.005	0.009 (±0.002)									
**G3**	0.030 ± 0.004	0.028 ± 0.004	0.010 (±0.002)								
**G4**	0.037 ± 0.005	0.035 ± 0.005	0.039 ± 0.005	0.011 (±0.002)							
**G5**	0.046 ± 0.006	0.047 ± 0.006	0.050 ± 0.006	0.037 ± 0.005	0.012 (±0.002)						
**G6**	0.041 ± 0.005	0.042 ± 0.005	0.042 ± 0.005	0.035 ± 0.005	0.045 ± 0.006	0.014 (±0.002)					
**G7**	0.040±0.005	0.039 ± 0.005	0.042 ± 0.006	0.029 ± 0.004	0.045 ± 0.006	0.041 ± 0.005	0.009 (±0.002)				
**G8**	0.023 ± 0.004	0.026 ± 0.004	0.027 ± 0.004	0.033 ± 0.005	0.045 ± 0.006	0.034 ± 0.005	0.036 ± 0.005	0.009 (±0.002)			
**G9**	0.023 ± 0.004	0.022 ± 0.004	0.022 ± 0.004	0.031 ± 0.005	0.043 ± 0.006	0.036 ± 0.005	0.035 ± 0.005	0.019 ± 0.004	0.001 (±0.001)		
**G10**	0.043 ± 0.005	0.044 ± 0.006	0.046 ± 0.006	0.036 ± 0.005	0.047 ± 0.006	0.031 ± 0.003	0.040 ± 0.005	0.039 ± 0.005	0.038 ± 0.006	0.010 (±0.002)	
**G12**	0.038 ± 0.006	0.039 ± 0.006	0.041 ± 0.004	0.025 ± 0.005	0.038 ± 0.006	0.037 ± 0.006	0.027 ± 0.005	0.034 ± 0.006	0.033 ± 0.006	0.039 ± 0.006	0.001 (±0.001)

## Data Availability

The data presented in this study are openly available in GeneBank (for accession numbers see Table S1).

## Supplementary Materials

The following are available online at https://www.mdpi.com/article/10.3390/pathogens11020180/s1, Figure S1: Phylogenetic analysis by the maximum likelihood method of a 400 bp fragment of *env* gene nucleotide sequences of Kazakhstan BLV isolates and known 11 BLV genotypes., Figure S2: SDT color-coded matrix of pairwise identity scores generated by the alignment of a G4, G7 and G12 400 bp long BLV *env* gene set of nucleotide sequences for 41 Kazakh BLV isolates and FLK-BLV strain., Figure S3: Phylogenetic tree of the deduced amino acid sequence. Table S1: Identity and origin of the sequences analyzed in the study. Table S2: BLV genotype reference sequences. Table S3 Pairwise identity scores of BLV sequences of cattle from Kazakhstan. Table S4 Amino acids substitutions in BLV ENV glycoprotein.
