# Supplementary material for: Molecular Characterization of Bovine Leukemia Virus with the Evidence of a New Genotype Circulating in Cattle from Kazakhstan
Source: Pathogens. 2022 Jan 28;11(2):180. doi: 10.3390/pathogens11020180 (PMC8875264; doi:10.3390/pathogens11020180)
Supplement: Supplementary file 1 [file pathogens-11-00180-s001.zip › Figure_S1_Phylogenetic analysis by ML of a 400 bp fragment of env gene .pdf]

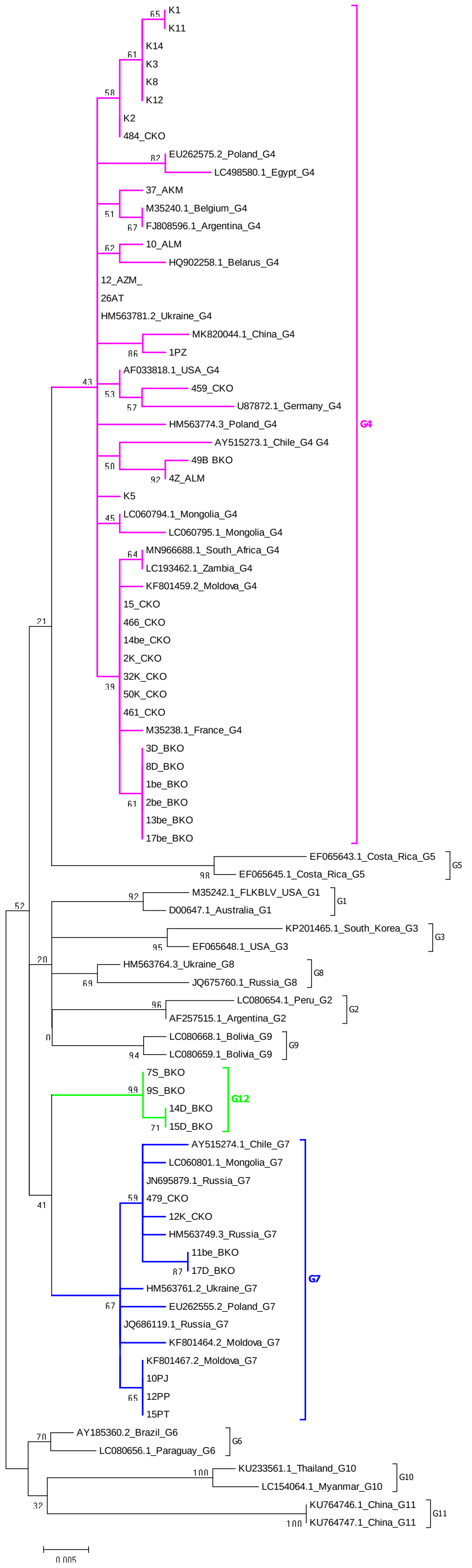

Figure S1. Phylogenetic analysis by the maximum likelihood method of a 400 bp fragment of *env* gene nucleotide sequences of Kazakhstan BLV isolates and known 11 BLV genotypes. The evolutionary history was inferred by using the Maximum Likelihood method based on the Kimura 2-parameter model. There were a total of 400 positions in the final dataset. Evolutionary analyses were conducted in MEGA6.
