# Supplementary material for: Molecular Characterization of Bovine Leukemia Virus with the Evidence of a New Genotype Circulating in Cattle from Kazakhstan
Source: Pathogens. 2022 Jan 28;11(2):180. doi: 10.3390/pathogens11020180 (PMC8875264; doi:10.3390/pathogens11020180)
Supplement: Supplementary file 1 [file pathogens-11-00180-s001.zip › Figure_S2._SDT color-coded matrix of pairwise identity score 400 bp BLV env seq.pdf]

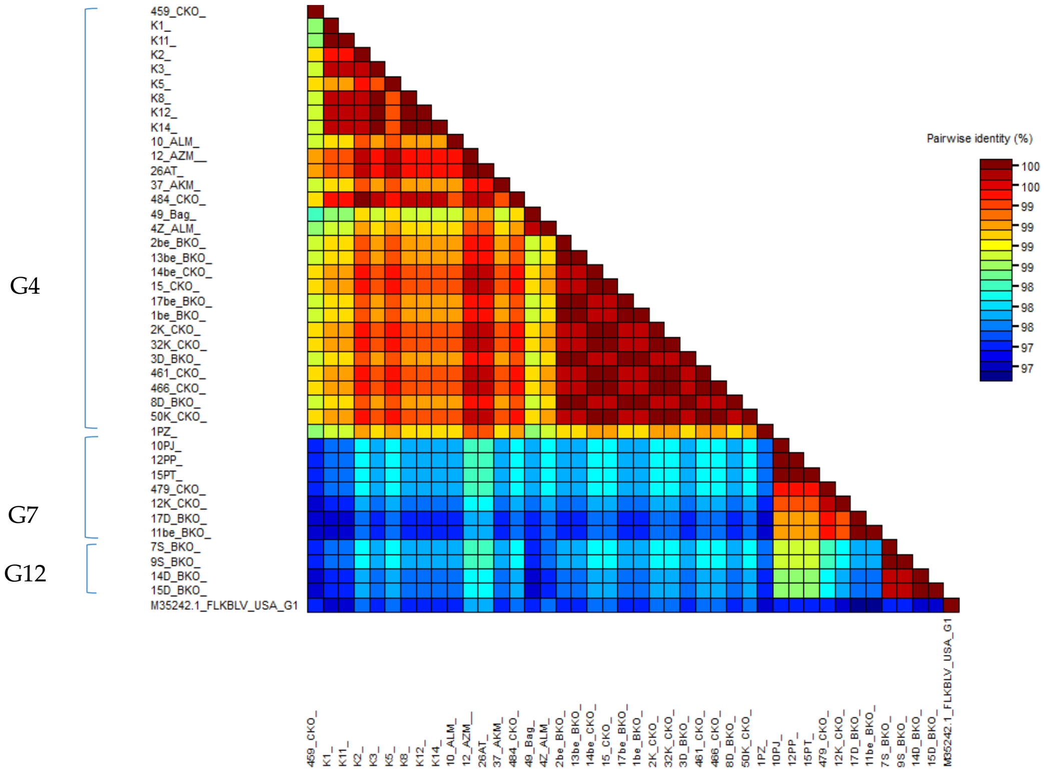

Figure S2. SDT colour-coded matrix of pairwise identity scores generated by the alignment of a G4, G7 and G12 400 bp long BLV *env* gene set of nucleotide sequences for 41 Kazakh BLV isolates and FLK-BLV strain
