# Supplementary material for: Molecular Characterization of Bovine Leukemia Virus with the Evidence of a New Genotype Circulating in Cattle from Kazakhstan
Source: Pathogens. 2022 Jan 28;11(2):180. doi: 10.3390/pathogens11020180 (PMC8875264; doi:10.3390/pathogens11020180)
Supplement: Supplementary file 1 [file pathogens-11-00180-s001.zip › Figure_S3_Phylogenetic tree of the deduced amino acid seq.pdf]

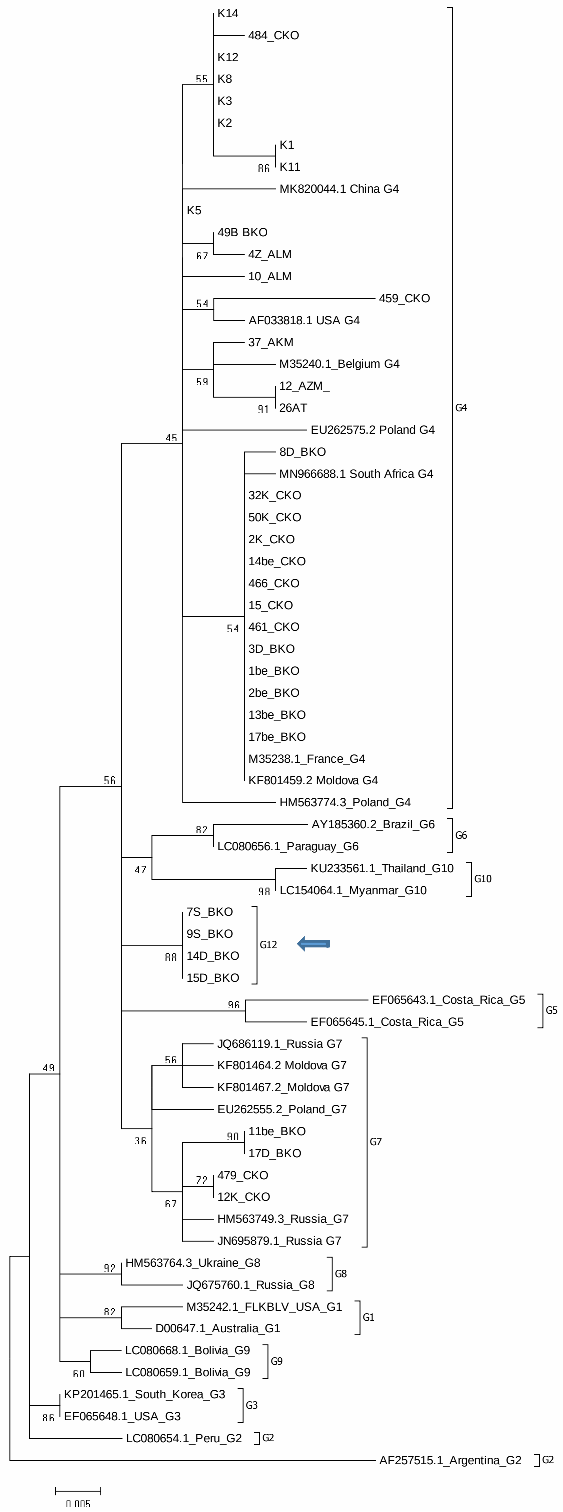

Figure S3:The evolutionary history was inferred by using the Maximum Likelihood method based on the JTT matrix-based model (MEGA6). Initial tree(s) for the heuristic search were obtained by applying the BioNJ method to a matrix of pairwise distances estimated using a JTT model. A discrete Gamma distribution was used to model evolutionary rate differences among sites (5 categories (+*G*, parameter = 0.7322)). The tree is drawn to scale, with branch lengths measured in the number of substitutions per site. The analysis involved 67 amino acid sequences. The coding data was translated assuming a Standard genetic code table. There were a total of 301 positions in the final dataset.
