# Supplementary material for: Molecular Characterization of Bovine Leukemia Virus with the Evidence of a New Genotype Circulating in Cattle from Kazakhstan
Source: Pathogens. 2022 Jan 28;11(2):180. doi: 10.3390/pathogens11020180 (PMC8875264; doi:10.3390/pathogens11020180)
Supplement: Supplementary file 1 [file pathogens-11-00180-s001.zip › Table S1 Identity and origin of the sequences analyzed in the study.pdf]

Table S1. Identity and origin of the sequences analysed in the study.

GenBank Accession No[1]	Name/Geographic origin	Genotype	Reference
OK631885	1PZ/Pavlodar	4	This paper
OK631886	10PJ/Pavlodar	7	This paper
OK631887	12PP/Pavlodar	7	This paper
OK631888	15PK/Pavlodar	7	This paper
OK945955	K1/Kostanay	4	This paper
OK945956	K2/Kostanay	4	This paper
OK945957	K3/Kostanay	4	This paper
OK945958	K5/Kostanay	4	This paper
OK945959	K8/Kostanay	4	This paper
OK945960	K11/Kostanay	4	This paper
OK945961	K12/Kostanay	4	This paper
OK945962	K14/Kostanay	4	This paper
OK945963	15_CKO/North Kazakhstan	4	This paper
OK945964	14be_CKO/ North Kazakhstan	4	This paper
OK945965	461_CKO/North Kazakhstan	4	This paper
OK945966	466_CKO/North Kazakhstan	4	This paper
OK945967	479_CKO/North Kazakhstan	7	This paper
OK945968	484_CKO/North Kazakhstan	4	This paper
OK945969	2K_CKO/North Kazakhstan	4	This paper
OK945970	12K_CKO/North Kazakhstan	7	This paper
OK945971	32K_CKO/North Kazakhstan	4	This paper
OK945972	50K_CKO/North Kazakhstan	4	This paper
OK945973	49_Bag/Bagration/East Kazakhstan	4	This paper
OK945974	7S_BKO/ Sredigornoje/East Kazakhstan	12	This paper
OK945975	9S_BKO/ Sredigornoje/East Kazakhstan	12	This paper
OK945976	14D_BKO/ Dorodnica/East Kazakhstan	12	This paper
OK945977	15D_BKO/ Dorodnica/East Kazakhstan	12	This paper
OK945978	3D_BKO/ Dorodnica/East Kazakhstan	4	This paper
OK945979	8D_BKO/ Dorodnica/East Kazakhstan	4	This paper
OK945980	17D_BKO/Dorodnica/ East Kazakhstan	7	This paper
OK945981	1be_BKO/ East Kazakhstan	4	This paper
OK945982	2be_BKO/ East Kazakhstan	4	This paper
OK945983	11be_BKO/ East Kazakhstan	7	This paper
OK945984	13be_BKO/ East Kazakhstan	4	This paper
OK945985	17be_BKO/ East Kazakhstan	4	This paper
OK945986	12_AZM/Almaty	4	This paper
OK945987	10_ALM/Almaty	4	This paper
OK945988	4Z_ALM/Almaty	4	This paper
OK945989	26_AT/Almaty	4	This paper
OK945990	37_AKM/Almaty	4	This paper
OK945991	459_CKO/North Kazakhstan	4	This paper
M35242.1	FLKBLV/USA	1	[1]
D00647.1	pBLV-A1/Australia	1	[2]
LC080654.1	lima40/Peru	2	[3]
AF257515.1	Argentina	2	[4]

KP201465.1	GBGS-12/South_Korea	3	[5]
EF065648.1	USCA-2/USA	3	[6]
M35238.1	LB59/France	4	[1]
KF801459.2	8MD/Moldova	4	[7]
EU262575.1	301_PL/Poland	4	[8]
HM563774.1	68_PL/Poland	4	[8]
M35240.1	LB285/Belgium	4	[1]
LC498580	DZ1.4/Egypt	4	[9]
MN966688.1	K1170/South Africa	4	[10]
LC060794.1	BLV_Mongolia-3/Mongolia	4	[11]
LC060795.1	BLV_Mongolia-4/Mongolia	4	[11]
HQ902258.1	1_BY/Belarus	4	[8]
MG204552.1	IR-Esfehan/Iran	4	[12]
HM563781	4_2_UA/Ukraine	4	[8]
AY515273	96/Chile	4	[13]
AF033818	USA	4	Direct submission by Chappey (1997)
FJ808596.1	LS-SFB/Argentina	4	[14]
LC193462.1	BLV-Zambia01-2016/Zambia	4	[15]
MK820044.1	B3008/China	4	[16]
U87872.1	3/Germany	4	[17]
EF065643.1	CRLV/Costa_Rica	5	[6]
EF065645.1	CRAG-1/Costa_Rica	5	[6]
AY185360.2	151/Brazil	6	[18]
LC080656	par62/Paraguay	6	[3]
AY515274.1	14/Chile	7	[13]
HM563761	3_1_UA/Ukraine	7	[8]
HM563749	5_RU/Russia	7	[8]
EU262555.1	151_PL/Poland	7	[8]
JN695879.1	9V/Russia,Vologda	7	Direct submission by Lomakina (2014)
JQ686119.1	NK13/Russia,Novosibirsk	7	Direct submission by Lomakina (2013)
S83530.1	I2/Italy	7	[19]
LC060801.1	BLV_Mongolia-10/Mongolia	7	[11]
KF801467	1MD/Moldova	7	[7]
KF801464	14MD/Moldova	7	[7]
HM563764.3	4-6/Ukraine	8	[8]
JQ675760.1	pr72/Russia	8	Direct submission by Lomakina (2013)
LC080668.1	por28/Bolivia	9	[3]
LC080659.1	mon1/Bolivia	9	[3]
KU233561.1	Thai_Saraburi_I_Thailand	10	[20]
LC154064.1	L2/ Myanmar	10	[21]
KU764746.1	E101/China	11	[22]
KU764747.1	E102/China	11	[22]

References:

1. Mamoun, R.Z.; Morisson, M.; Rebeyrotte, N.; Busetta, B.; Couez, D.; Kettmann, R.; Hospital, M.; Guillemain, B. Sequence variability of bovine leukemia virus env gene and its relevance to the

structure and antigenicity of the glycoproteins. *J. Virol.* **1990**, *64*, 4180–4188, doi:10.1128/jvi.64.9.4180-4188.1990.

2. Coulston, J.; Naif, H.; Brandon, R.; Kumar, S.; Khan, S.; Daniel, R.C.W.; Lavin, M.F. Molecular Cloning and Sequencing of an Australian Isolate of Proviral Bovine Leukaemia Virus DNA: Comparison with other Isolates. *J. Gen. Virol.* **1990**, *71*, 1737–1746, doi:10.1099/0022-1317-71-8-1737.
3. Polat, M.; Takeshima, S.; Hosomichi, K.; Kim, J.; Miyasaka, T.; Yamada, K.; Arainga, M.; Murakami, T.; Matsumoto, Y.; de la Barra Diaz, V.; et al. A new genotype of bovine leukemia virus in South America identified by NGS-based whole genome sequencing and molecular evolutionary genetic analysis. *Retrovirology* **2016**, *13*, 4, doi:10.1186/s12977-016-0239-z.
4. Dube, S.; Dolcini, G.; Abbott, L.; Mehta, S.; Dube, D.; Gutierrez, S.; Ceriani, C.; Esteban, E.; Ferrer, J.; Poiesz, B. The Complete Genomic Sequence of a BLV Strain from a Holstein Cow from Argentina. *Virology* **2000**, *277*, 379–386, doi:10.1006/viro.2000.0622.
5. Lee, E.; Kim, E.-J.; Joung, H.-K.; Kim, B.-H.; Song, J.-Y.; Cho, I.-S.; Lee, K.-K.; Shin, Y.-K. Sequencing and phylogenetic analysis of the gp51 gene from Korean bovine leukemia virus isolates. *Virol. J.* **2015**, *12*, 64, doi:10.1186/s12985-015-0286-4.
6. Zhao, X.; Buehring, G.C. Natural genetic variations in bovine leukemia virus envelope gene: Possible effects of selection and escape. *Virology* **2007**, *366*, 150–165, doi:10.1016/j.virol.2007.03.058.
7. Pluta, A.; Rola-Łuszczak, M.; Kubiś, P.; Balov, S.; Moskalik, R.; Choudhury, B.; Kuźmak, J. Molecular characterization of bovine leukemia virus from Moldovan dairy cattle. *Arch. Virol.* **2017**, *162*, 1563–1576, doi:10.1007/s00705-017-3241-4.
8. Rola-Łuszczak, M.; Pluta, A.; Olech, M.; Donnik, I.; Petropavlovskiy, M. The Molecular Characterization of Bovine Leukaemia Virus Isolates from Eastern Europe and Siberia and Its Impact on Phylogeny. *PLoS One* **2013**, *8*, 58705, doi:10.1371/journal.pone.0058705.
9. Hamada, R.; Metwally, S.; Polat, M.; Borjigin, L.; Ali, A.O.; Abdel-Hady, A.A.A.; Mohamed, A.E.A.; Wada, S.; Aida, Y. Detection and Molecular Characterization of Bovine Leukemia Virus in Egyptian Dairy Cattle. *Front. Vet. Sci.* **2020**, *7*, doi:10.3389/fvets.2020.00608.
10. Suzuki, A.; Chapman, R.; Douglass, N.; Carulei, O.; van Rensburg, J.; Williamson, A.-L. Phylogenetic Analysis of South African Bovine Leukaemia Virus (BLV) Isolates. *Viruses* **2020**, *12*, 898, doi:10.3390/v12080898.
11. Ochirkhuu, N.; Konnai, S.; Odbileg, R.; Nishimori, A.; Okagawa, T.; Murata, S.; Ohashi, K. Detection of bovine leukemia virus and identification of its genotype in Mongolian cattle. *Arch. Virol.* **2016**, *161*, 985–991, doi:10.1007/s00705-015-2676-8.
12. Kazemimanesh, M.; Madadgar, O.; Steinbach, F.; Choudhury, B.; Azadmanesh, K. Detection and molecular characterization of bovine leukemia virus in various regions of Iran. *J. Gen. Virol.* **2019**, *100*, 1315–1327, doi:10.1099/jgv.0.001303.
13. Felmer, R.; Muñoz, G.; Zúñiga, J.; Recabal, M. Molecular analysis of a 444bp fragment of the bovine leukaemia virus gp51 env gene reveals a high frequency of non-silent point mutations and suggests the presence of two subgroups of BLV in Chile. *Vet. Microbiol.* **2005**, *108*, 39–47, doi:10.1016/j.vetmic.2005.04.005.
14. Rodriguez, S.M.; Golemba, M.D.; Campos, R.H.; Trono, K.; Jones, L.R. Bovine leukemia virus can be classified into seven genotypes: evidence for the existence of two novel clades. *J. Gen. Virol.* **2009**, *90*, 2788–2797, doi:10.1099/vir.0.011791-0.
15. Pandey, G.S.; Simulundu, E.; Mwiinga, D.; Samui, K.L.; Mweene, A.S.; Kajihara, M.; Mangani, A.; Mwenda, R.; Ndebe, J.; Konnai, S.; et al. Clinical and subclinical bovine leukemia virus infection in a dairy cattle herd in Zambia. *Arch. Virol.* **2017**, *162*, 1051–1056, doi:10.1007/s00705-016-3205-0.
16. Yang, Y.; Chen, L.; Dong, M.; Huang, W.; Hao, X.; Peng, Y.; Gong, Z.; Qin, A.; Shang, S.; Yang, Z. Molecular characterization of bovine leukemia virus reveals existence of genotype 4 in Chinese dairy cattle. *Virol. J.* **2019**, *16*, 108, doi:10.1186/s12985-019-1207-8.

17. Fechner, H.; Blankenstein, P.; Looman, A.C.; Elwert, J.; Geue, L.; Albrecht, C.; Kurg, A.; Beier, D.; Marquardt, O.; Ebner, D. Provirus Variants of the Bovine Leukemia Virus and Their Relation to the Serological Status of Naturally Infected Cattle. *Virology* **1997**, *237*, 261–269, doi:10.1006/viro.1997.8784.
18. Camargos, M.F.; Pereda, A.; Stancek, D.; Rocha, M.A.; Reis, J.K.P. dos; Greiser-Wilke, I.; Leite, R.C. Molecular characterization of the env gene from Brazilian field isolates of Bovine Leukemia Virus. *Virus Genes* **2007**, *34*, 343–350, doi:10.1007/s11262-006-0011-x.
19. Molteni, E.; Agresti, A.; Meneveri, R.; Marozzi, A.; Malcovati, M.; Bonizzi, L.; Poli, G.; Ginelli, E. Molecular Characterization of a Variant of Proviral Bovine Leukaemia Virus (BLV). *J. Vet. Med. Ser. B* **1996**, *43*, 201–211, doi:10.1111/j.1439-0450.1996.tb00307.x.
20. Lee, E.; Kim, E.-J.; Ratthanophart, J.; Vitoonpong, R.; Kim, B.-H.; Cho, I.-S.; Song, J.-Y.; Lee, K.-K.; Shin, Y.-K. Molecular epidemiological and serological studies of bovine leukemia virus (BLV) infection in Thailand cattle. *Infect. Genet. Evol.* **2016**, *41*, 245–254, doi:10.1016/j.meegid.2016.04.010.
21. Polat, M.; Moe, H.H.; Shimogiri, T.; Moe, K.K.; Takeshima, S.; Aida, Y. The molecular epidemiological study of bovine leukemia virus infection in Myanmar cattle. *Arch. Virol.* **2017**, *162*, 425–437, doi:10.1007/s00705-016-3118-y.
22. Yu, C.; Wang, X.; Zhou, Y.; Wang, Y.; Zhang, X.; Zheng, Y. Genotyping bovine leukemia virus in dairy cattle of Heilongjiang, northeastern China. *BMC Vet. Res.* **2019**, *15*, 179, doi:10.1186/s12917-019-1863-3.
