# Supplementary material for: Molecular Characterization of Bovine Leukemia Virus with the Evidence of a New Genotype Circulating in Cattle from Kazakhstan
Source: Pathogens. 2022 Jan 28;11(2):180. doi: 10.3390/pathogens11020180 (PMC8875264; doi:10.3390/pathogens11020180)
Supplement: Supplementary file 1 [file pathogens-11-00180-s001.zip › Table S2 BLV_genotype_reference_sequences.pdf]

Table S2. BLV genotype reference sequences

No	Accession	Genotype
1	KU233564.1	G_1
2	LC512446.1	G_1
3	LC512445.1	G_1
4	LC361266.1	G_1
5	MW926774.1	G_1
6	LC080651.1	G_1
7	LC007992.1	G_1
8	MW926773.1	G_1
9	MW926779.1	G_1
10	MW926775.1	G_1
11	MW926772.1	G_1
12	LC080653.1	G_1
13	LC007990.1	G_1
14	LC007983.1	G_1
15	LC361265.1	G_1
13	LC007991.1	G_1
17	LC080652.1	G_1
18	LC361264.1	G_1
19	MW926778.1	G_1
20	MW926782.1	G_1
21	MG678789.1	G_1
22	MH170027.1	G_1
23	FM209468.1	G_1
24	AB934282.1	G_1
25	MW926783.1	G_1
26	FM209469.1	G_1
27	MG678787.1	G_1
28	MN966692.1	G_1
29	MW926776.1	G_1
30	MG678786.1	G_1
31	MH170028.1	G_1
32	MG678788.1	G_1
33	MG678785.1	G_1
34	LC361255.1	G_1
35	LC361256.1	G_1
36	MG678760.1	G_1
37	LC361254.1	G_1
38	LC361262.1	G_1
39	LC361263.1	G_1
40	LC361258.1	G_1
41	LC512447.1	G_1
42	LC512448.1	G_1
43	MG678763.1	G_1
44	KU233556.1	G_1
45	MG678761.1	G_1
46	MG678762.1	G_1
47	MG678759.1	G_1
48	LC361257.1	G_1
49	LC007988.1	G_1
50	LC007987.1	G_1
51	LC361261.1	G_1
52	LC007989.1	G_1
53	LC007981.1	G_1
54	LC007980.1	G_1
55	LC007985.1	G_1
56	LC361249.1	G_1
57	LC361250.1	G_1
58	LC361253.1	G_1
59	LC361248.1	G_1
60	LC361251.1	G_1
61	LC361252.1	G_1
62	LC361260.1	G_1
63	MG678775.1	G_1
64	MG678774.1	G_1
65	MG678770.1	G_1
66	MG678773.1	G_1
67	KP201481.1	G_1
68	KP201482.1	G_1
69	MG678768.1	G_1
70	MG678782.1	G_1
71	MG678783.1	G_1
72	MG678780.1	G_1
73	MG678781.1	G_1
74	MG678771.1	G_1
75	MG678772.1	G_1
76	MG678776.1	G_1
77	MG678767.1	G_1
78	LC007977.1	G_1
79	LC007978.1	G_1
80	LC007979.1	G_1
81	KU233565.1	G_1
82	KU233557.1	G_1
83	KU233558.1	G_1
84	KU233559.1	G_1
85	MG678766.1	G_1
86	MG678765.1	G_1
87	MG678764.1	G_1
88	LC005615.1	G_1
89	LC005616.1	G_1
90	KU233566.1	G_1
91	KU233567.1	G_1
92	HE967302.1	G_1
93	HE967301.1	G_1
94	FM209472.1	G_1
95	MG678784.1	G_1
96	KP201473.1	G_1
97	KP201472.1	G_1
98	KP201471.1	G_1
99	FM209470.1	G_1
100	MW926784.1	G_1
101	MW926785.1	G_1
102	MW926777.1	G_1
103	HE967303.1	G_1
104	MW926780.1	G_1
105	MW926781.1	G_1
106	KP201470.1	G_1
107	KP201476.1	G_1
108	KP201475.1	G_1
109	KP201474.1	G_1
110	MG678769.1	G_1
111	MG678777.1	G_1
112	MG678778.1	G_1
113	MG678779.1	G_1
114	KP201469.1	G_1
115	KP201468.1	G_1
116	KP201467.1	G_1
117	KP201478.1	G_1
118	KP201477.1	G_1
119	KP201480.1	G_1
120	KP201479.1	G_1
121	FJ914764.1	G_2
122	LC080655.1	G_2
123	LC080654.1	G_2
124	AF399704.3	G_2
125	LC361267.1	G_3
126	MG678790.1	G_3
127	KP201465.1	G_3
128	LC361259.1	G_3
129	KP201464.1	G_3
130	JQ686096.1	G_4
131	JQ686098.1	G_4
132	JQ686093.1	G_4
133	MN966690.1	G_4
134	MN966691.1	G_4
135	JQ686090.1	G_4
136	MN966693.1	G_4
137	JQ686117.1	G_4
138	JQ686106.1	G_4

139	JQ686107.1	G_4
140	JQ686112.1	G_4
141	JQ686095.1	G_4
142	JQ686094.1	G_4
143	JQ686111.1	G_4
144	JQ686089.1	G_4
145	MN966695.1	G_4
146	MK840879.1	G_4
147	JQ686091.1	G_4
148	MN966689.1	G_4
149	JQ686118.1	G_4
150	MN966694.1	G_4
151	MN966688.1	G_4
152	JQ675758.1	G_4
153	AF067081.1	G_4
154	AF503581.1	G_4
155	HM563774.3	G_4
156	JQ686092.1	G_4
157	MK840877.1	G_4
158	MK820044.1	G_4
159	EF065635.1	G_5
160	EF065639.1	G_5
161	EF065643.1	G_5
162	EF065645.1	G_5
163	EF065654.1	G_5
164	EF065655.1	G_5
165	MW926786.1	G_6
166	MW926789.1	G_6
167	LC080658.1	G_6
168	MW926792.1	G_6
169	MW926791.1	G_6
170	MW926794.1	G_6
171	MW926788.1	G_6
172	MW926787.1	G_6
173	MW926793.1	G_6
174	LC080657.1	G_6
175	MK840880.1	G_6
176	LC080656.1	G_6
177	MK840878.1	G_6
178	KU233563.1	G_6
179	MK840876.1	G_6
180	MK840875.1	G_6
181	KU233562.1	G_6
182	MH341525.1	G_6
183	MH341521.1	G_6
184	MH341524.1	G_6
185	MH341516.1	G_6
186	MH341522.1	G_6
187	MH341523.1	G_6
188	MH341520.1	G_6
189	MH341519.1	G_6
190	MH341518.1	G_6
191	MH341517.1	G_6
192	LC512450.1	G_6
193	MH170030.1	G_6
194	MW926790.1	G_6
195	LC512449.1	G_6
196	MH170029.1	G_6
197	LC512451.1	G_6
198	KU233548.1	G_6
199	KU233549.1	G_6
200	KU233544.1	G_6
201	KF801470.2	G_7
202	KF801469.2	G_7
203	KF801467.2	G_7
204	HM563758.3	G_7
205	KF801468.2	G_7
206	HM563749.3	G_7
207	JQ675756.1	G_7
208	JQ675757.1	G_7
209	KF801466.2	G_7
210	JQ686120.1	G_7
211	JQ686116.1	G_7
212	JQ686119.1	G_7
213	JQ675760.1	G_8
214	JQ675759.1	G_8
215	HM563764.3	G_8
216	LC080663.1	G_9
217	LC080662.1	G_9
218	LC080659.1	G_9
219	LC080664.1	G_9
220	LC080674.1	G_9
221	LC080668.1	G_9
222	LC080666.1	G_9
223	LC080675.1	G_9
224	LC080660.1	G_9
225	LC080667.1	G_9
226	LC080673.1	G_9
227	LC080670.1	G_9
228	LC080669.1	G_9
229	LC080665.1	G_9
230	LC080661.1	G_9
231	LC080672.1	G_9
232	LC080671.1	G_9
233	KU233554.1	G_10
234	LC154064.1	G_10
235	KU233551.1	G_10
236	KU233555.1	G_10
237	LC154066.1	G_10
238	LC154065.1	G_10
239	LC154067.1	G_10
240	LC154848.1	G_10
241	LC512452.1	G_10
242	KU233545.1	G_10
243	KU233543.1	G_10
244	KU233552.1	G_10
245	KU233553.1	G_10
246	KU233550.1	G_10
247	LC154847.1	G_10
248	KU233549.1	G_10
249	KU233546.1	G_10
250	KU233561.1	G_10
251	7S_BKO	G_12
252	14D_BKO	G_12
253	15D_BKO	G_12
254	9S_BKO	G_12
