# Supplementary material for: Molecular Characterization of Bovine Leukemia Virus with the Evidence of a New Genotype Circulating in Cattle from Kazakhstan
Source: Pathogens. 2022 Jan 28;11(2):180. doi: 10.3390/pathogens11020180 (PMC8875264; doi:10.3390/pathogens11020180)
Supplement: Supplementary file 1 [file pathogens-11-00180-s001.zip › Table_S4_Amino_acids_substitutions_In_BLV_ENV-2.pdf]

Number	Genotype	7	12	15	24	28	29	32	33	37	48	56	58	73	74	80	82	106	121	132	133	134	137	140	141	143	144	177	189	252	264	267	290	291	301
4	12	S	Q	I	L	C	Q	Q	A	S	T	S	S	A	R	L	F	V	R	Q	A	D	S	I	N	Q	I	P	S	T	P	R	T	A	R
18	10								T									A	6H				17F	V	1S	13R	T	S							11P
17	9								T		A				K		12L				V			V											
3	8						P		T		A				K									V								K			
12	7	6P/1F						4R	T		5I													11V/1I										**V**	
36	6	1P	E						T															V	1D		T		7N	10A		K		2V	10P
6	5			V	V		2R		T	4T						S		1I		R	1T			V									5M	5T	
29	4								T			13F	6A	1L/28P		1S			27H	7R	1P			V			13T								
5	3								T						K	2M					T			V	D										
4	2								T						K									V	D										
120	1	1P	2P	2T		1F/42S	119R		T						K		S	4A	2H/1L	1R	1T	11N	1A	5A/115V		1H	1T/2N		9R	1P	S			2V/1T	12P/1G

Table S4 Amino acid substitutions in BLV ENV glycoprotein of Kazakhstan genotype G12 sequences and 250 sequences representative for G1-G10 genotypes. Kazakhstan BLV sequences clustered as genotype G4 and G7 were not included in the analysis. Particular parsimony informative sites were selected based on the occurrence of amino acid substitution characteristic for genotype/genotypes. The number next to the amino acids in each position indicates the number of given substitution in that genotypes. The presence of one amino acid indicates that it was present in 100% of the sequences of the genotype. Blank spaces - amino acid identical to genotype G12.
